# Randomized Clinical Trial Comparing Concomitant and Tailored Therapy for Eradication of *Helicobacter pylori* Infection

**DOI:** 10.3390/jpm11060534

**Published:** 2021-06-09

**Authors:** Nikola Perkovic, Antonio Mestrovic, Josko Bozic, Mirela Pavicic Ivelja, Jonatan Vukovic, Goran Kardum, Zeljko Sundov, Marija Tonkic, Zeljko Puljiz, Katarina Vukojevic, Ante Tonkic

**Affiliations:** 1 Department of Gastroenterology, University Hospital of Split, 21000 Split, Croatia; antonio.mestrovic1@gmail.com (A.M.); jvukovic@mefst.hr (J.V.); zsundov@gmail.com (Z.S.); zpuljiz4@gmail.com (Z.P.); ante.tonkic@mefst.hr (A.T.); 2 Department of Pathophysiology, University of Split School of Medicine, 21000 Split, Croatia; josko.bozic@mefst.hr; 3 Department for Infectious Diseases, University Hospital of Split, 21000 Split, Croatia; mmarendic@gmail.com; 4 Department of Psychology, Faculty of Humanities and Social Sciences, University of Split, 21000 Split, Croatia; goran.kardum@unist.hr; 5 Department of Internal Medicine, University of Split School of Medicine, 21000 Split, Croatia; 6 Department of Microbiology and Parasitology, University Hospital of Split, 21000 Split, Croatia; mtonkic@kbsplit.hr; 7 Department of Medical Microbiology and Parasitology, University of Split School of Medicine, 21000 Split, Croatia; 8 Department of Anatomy, University of Split School of Medicine, 21000 Split, Croatia; kvukojev@gmail.com

**Keywords:** *H. pylori*, concomitant therapy, tailored therapy, gastritis, antimicrobial resistance

## Abstract

As high clarithromycin resistance (>20%) in the Split-Dalmatia region of Croatia hinders the treatment of *H. pylori* infection, the primary objective of this study was to compare concomitant quadruple with the tailored, personalized therapy as first-line eradication treatment of *H. pylori*. In an open-label, randomized clinical trial, 80 patients with *H. pylori* infection were randomly assigned to either concomitant (esomeprazole 40 mg, amoxicillin 1 gr, metronidazole 500 mg, clarithromycin 500 mg, twice daily for 14 days) or tailored therapy in accordance with the results of the antimicrobial susceptibility testing. Eradication status was assessed 4 weeks after treatment. Eradication rates were significantly higher in tailored group than in concomitant group both in intention-to-treat (70 vs. 92.5%, *p* = 0.010) and per-protocol (87.5 vs. 100%, *p* = 0.030) analysis in the setting of increasing antibiotic resistance (clarithromycin 37.5%, metronidazole 17.5%, dual resistance 10%). Adverse effects were more frequent in the concomitant group (32.5 vs. 7.5%, *p* = 0.006). Tailored therapy achieves higher eradication with a lower adverse events rate. With the increasing resistance of *H. pylori* strains to antibiotic treatment, eradication regimes with such characteristics should be strongly considered as a reasonable choice for first-line treatment.

## 1. Introduction

More than half of the world’s population are *H. pylori* carriers [1]. The infection is mostly acquired in childhood and persists lifelong. A notable risk factor is a lower social and economic status during childhood, reflecting mostly poor hygienic standards or small and dense living areas [2]. Newly acquired infections in adulthood are a rarity. The reservoir of *H. pylori* is the human stomach. *H. pylori* is considered to be the main pathogen involved in causing benign peptic ulcers and functional dyspepsia, as well as gastric cancer [3,4]. It was shown that *H. pylori* contains cytosolic alcohol dehydrogenase (ADH) and consequently is capable of producing acetaldehyde from excess ethanol in vitro. *H. pylori* infection is associated with decreased alcohol dehydrogenase (ADH) activity in the gastric mucosa, which depends on the severity of inflammation and mucosal injury [5]. This damage can be a reason for the release of enzymes from gastric mucosa, which leads to the increase in the ADH activity in the sera of patients with *H. pylori* infection. Among all tested classes of ADH isoenzymes, only class IV had higher activity in the serum of patients with *H. pylori* infection, thereby presenting itself as a potential marker of the infection [6].

The 2015 Kyoto Consensus defined *H. pylori* gastritis as an infectious disease, requiring treatment regardless of symptomatology because eradication can prevent above mentioned complications [7].

The treatment of *H. pylori* infection is currently complicated by an increase in antimicrobial resistance in different parts of the world [8]. The corresponding increase in clarithromycin, as well as quinolone and metronidazole resistance, poses a major clinical problem and calls for a new approach to treatment [8,9]. Under such circumstances, there is an emerging trend towards personalized eradication therapy [10,11]. Since *H. pylori* infection is an infectious disease, its optimal treatment should both theoretically and practically be based on the specific characteristics of the strain, and if possible, the host of the infection [12,13]. The aim of such an approach should be a better eradication efficacy.

Non-bismuth quadruple therapies have been proposed as potential strategies in improving the efficacy of first-line treatments [14,15]. The non-bismuth quadruple therapy in its concomitant variant consists of proton pump inhibitors (PPI), amoxicillin, nitroimidazole and clarithromycin given concurrently twice daily. As a result of concurrent administration, this therapy has given better results according to some studies in comparison to sequential variants [16,17]. However, this therapy suffers from the aforementioned increase in antibiotic resistance as well. Furthermore, there is no defined optimal eradication therapy for *H. pylori* infection that would be equally effective in all regions [18,19]. Therefore, it is advised to determine primary resistance to commonly used antibiotics in the eradication of *H. pylori* infection in each region [20,21]. To our knowledge, the efficacy of tailored therapy in the treatment of *H. pylori* in Croatia has not been investigated to date. Given that the choice of eradication therapy is primarily based on local antibiotic resistance, we consider it is essential to examine the efficacy of tailored and concomitant therapy in *H. pylori* eradication in the Split-Dalmatia area, knowing that the clarithromycin resistance is above 20% in our region [22]. Therefore, the aim of our study was to compare concomitant nonbismuth quadruple therapy with a tailored therapy based on antibiotic strain susceptibility testing, assuming that the eradication rate with tailored therapy will be above 90%. Secondary aims were to establish the compliance and adverse events rate.

## 2. Materials and Methods

### 2.1. Design Overview

A prospective, open-label, randomized controlled trial was performed at the University Hospital of Split from January 2019 to January 2020. All patients with dyspeptic symptoms who were referred for upper endoscopy were included in the study. Patients were eligible if they were older than 18 and had a documented *H. pylori* infection according to the Maastricht V guidelines [23].

Patients with any one of the following criteria were excluded from the study: age less than 18 years; previously unsuccessful application of empirical *H. pylori* eradication therapy; malignant disease of the stomach or any other site; taking proton pump inhibitors (PPI), H2 antagonists, bismuths or antibiotics (amoxicillin, metronidazole, clarithromycin) during the last month; associated comorbidity (renal insufficiency, mental illness); drug allergies: proton pumps inhibitors or antibiotics (amoxicillin, metronidazole, clarithromycin); pregnancy and lactation; refusal to participate in the survey.

The study included both hospitalized and outpatient clinic patients that had *H. pylori* infection verified in one of these tests: positive stool antigen test (based on the monoclonal antibody, ELISA), positive rapid urease test during upper endoscopy, positive histology biopsy test or positive urea breath test. Patients with positive *H. pylori* finding were recruited in the study between January 2019 and January 2020. All patients had to have signed informed consent. They were interviewed by medical staff for medical history and demographic data and then randomly assigned to two groups via the computer-generated simple randomization scheme. Written instructions about the therapy timing and dosage were given to each of the participants. The protocol was approved by the ethics committee of the University of Split Hospital Centre (as from July 2018, approval number 500-03/18-01/59) and registered as a clinical trial (Clinical Trials, gov: NCT04621487). The authors confirm that all ongoing and related trials for this drug/intervention are registered. The study was conducted according to the principles of the Declaration of Helsinki and the standards of good clinical practice.

### 2.2. Biopsy Sampling and Microbiology Tests

*H. pylori* strains for antibiotic susceptibility testing were isolated from gastric mucosal samples (one from gastric antrum and one from gastric corpus) obtained during an upper endoscopy. They were cultured in the Department of Microbiology and Parasitology, University hospital of Split on Pylori agar (bioMerieux, Marcy l’Etoile, France) after incubation for 3–5 days, at 37 °C, in the microaerophilic atmosphere. The susceptibility of *H. pylori* isolates to amoxicillin, clarithromycin, tetracycline, levofloxacin and metronidazole was determined by an E-test (AB Biodisk, Solna, Sweden). E-tests were performed on Columbia agar plates with 7% horse blood without supplement. Plates were inoculated with bacterial suspension (turbidity of 3–4 McFarland) and incubated at 37 °C for 72 h under a microaerophilic atmosphere. The antibiotic breakpoints were >0.125 mg/L for amoxicillin, >0.5 mg/L for clarithromycin, >1 mg/L for tetracycline, >1 mg/L for levofloxacin, and >8 mg/L for metronidazole.

### 2.3. Patient Follow-Up

One month after ending the therapy, all patients had an ELISA-based stool *H. pylori* antigen test performed in the Department of Microbiology and parasitology of the University Hospital of Split. With the results of that, test patients were reassessed for therapeutic compliance and incidence of side effects. Eradication failure was defined as a positive result of this test. During the follow-up, compliance and adverse events were evaluated. The compliance was defined by the amount of medication taken (compliance was considered good if ≥80% of therapy was taken), based on the remaining pill count and the patient’s self-reported questionnaire that included information regarding compliance and adverse events.

The adverse events included: nausea, abdominal pain, diarrhea, constipation, dizziness, metal taste (in mouth), headache, loss of appetite, vomiting, skin rash, itching, black tongue and tongue deposits.

The adverse events were divided into groups according to the degree of tolerance: no adverse events; mild (without limitation in daily activities); moderate (partly limited daily activities); and severe (completely limited daily activities). Patients were instructed to report immediately in case of any severe adverse events.

The primary outcome of the study was to compare *H. pylori* eradication rates in patients receiving concomitant and tailored therapy. Secondary outcomes were the assessment of compliance and adverse events in both groups.

### 2.4. Therapy

The eligible participants were randomly assigned, using computer generating sequence in two groups. The first group was given concomitant therapy: esomeprazole 40 mg, amoxicillin 1 g, clarithromycin 500 mg and metronidazole 500 mg, which were all administered orally twice daily for a total of 14 days. The second group was given tailored therapy, which consisted of 14 days of antibiotic therapy according to *H. pylori* strains antibiotic sensitivity test together with esomeprazole 40 mg twice daily. The antibiotic therapy included two antimicrobial agents, of which the susceptibility testing was positive. Written instructions on the dose and timing of treatment were provided to each subject individually.

### 2.5. Statistical Analysis

#### 2.5.1. Sample Size Calculation

The total number of participants was calculated based on the effect size parameter (w = 0.3), statistical significance (*p* = 0.01), and power of 0.90. Based on the input parameters, a sample size of 40 subjects per group was required. Sample size calculations were made using the power analysis statistical package in the R interface (ver. 3.4.3, 2017).

#### 2.5.2. Statistical Analysis

Statistical software SPSS ver. 25 (IBM Corp, Armonk, NY, USA) and MedCalc statistical package (version 19.1.2, MedCalc Software, Ostend, Belgium) was used for statistical data analysis. Mean value and standard deviation (SD) or whole numbers with percentages were used for data description. Furthermore, 95% confidence intervals (CIs) were calculated for eradication rate variables. Chi-squared test with Yates’ correction or Fisher’s exact test was used for categorical data comparisons, while t-test was used for age comparison between groups. Binomial logistic regression analysis was employed to assess adjusted odds ratios (aOR) of adverse effects, while age and gender variables were used as covariates. The concomitant therapy group was set as a reference group. Analysis was performed by intention-to-treat (ITT) and per-protocol (PP). The ITT population included all randomized patients who received at least one dose of used drugs. The PP analysis excluded the patients with unknown *H. pylori* status following therapy (lost to follow-up) and patients with poor compliance to the therapy (<80%). All assumptions for the use of statistical tests have been fulfilled. The statistical significance was set at *p* < 0.05.

## 3. Results

### 3.1. Study Group Characteristics

**Figure 1:**
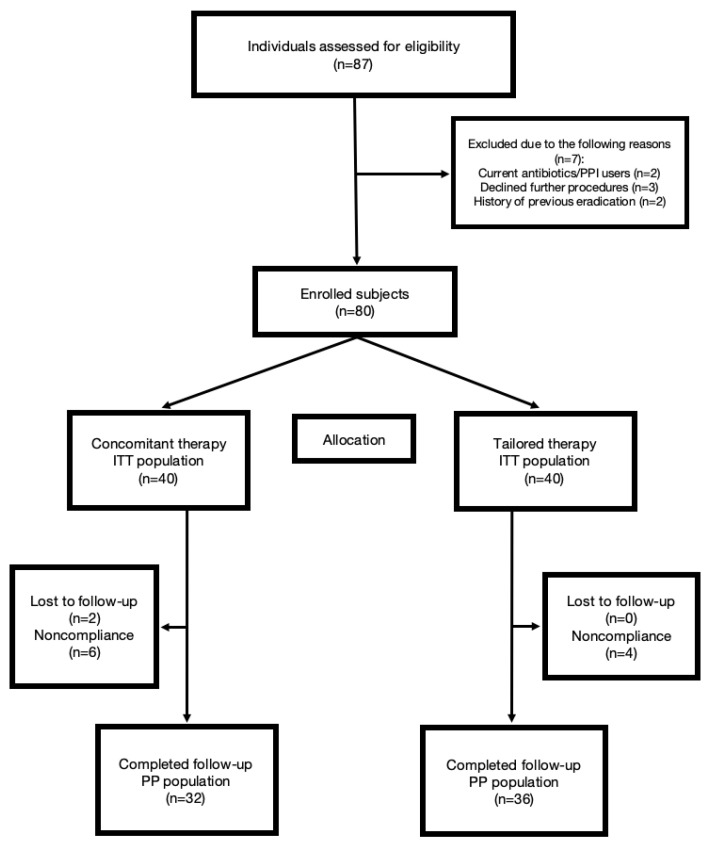
Study flowchart. PPI: proton pump inhibitors; ITT: intention-to-treat; PP: per-protocol.

Among 87 patients screened, seven were excluded due to screening failure. A total of 80 patients were randomly assigned to either the concomitant therapy (n = 40) or tailored therapy (n = 40) group. Table 1 shows the baseline characteristics of the included patients. There were no statistically significant differences between the two groups in terms of age, sex, history of smoking, alcohol use, or endoscopic finding. Five patients total in the concomitant group, and one patient in the tailored group was lost to follow-up. In each group, three patients consumed less than 80% of prescribed medications. A flowchart of the recruitment of study participants is shown in Figure 1.

### 3.2. Outcomes

#### 3.2.1. Eradication and Antimicrobial Resistance Rate

For the intention-to-treat (ITT) analysis, the eradication rates of *H. pylori* were 70% (28/40; 95% CI: 55.8–84.2) in the concomitant group and 90% (36/40; 95% CI: 85.0–100.0) in the tailored therapy group *p* = 0.010). For the per-protocol (PP) analysis, the eradication rates were 87.5% (28/32; 95% CI: 76.1–98.9) in the concomitant group and 100.0% (36/36;/) in the tailored group (*p* = 0.030) (Table 2). In total 40 strains were analyzed for antimicrobial susceptibility. The resistance rate among the strains was 37.5% (n = 15) to clarithromycin, 17.5% (n = 7) to metronidazole, 5% (n = 2) to levofloxacin and no strain was resistant to amoxicillin (Table 3). Dual resistance was detected to clarithromycin and metronidazole in 10% (n = 4) of strains and to clarithromycin and levofloxacin in 5% (n = 2) of strains. There were 52.5% (n = 21) of strains sensitive to all the antimicrobial drugs tested. Regarding antimicrobial drugs used for eradication treatment 52.5% (n = 21) were treated with amoxicillin and clarithromycin, 25% (n = 10) with amoxicillin and metronidazole, 15% (n = 6) with amoxicillin and levofloxacin and 7.5% (n = 3) with clarithromycin and metronidazole.

#### 3.2.2. Compliance and Adverse Events

There was no significant difference in the compliance rate between the two groups (*p* = 0.502). Six patients in the concomitant group and four patients in the tailored group had a compliance rate below 80%. Adverse events occurred significantly higher in concomitant than in tailored group (32.5% vs. 7.5%, *p* = 0.006) (Table 4). The tailored group also had significantly lower adjusted odds of adverse events (aOR 0.16, 95%CI 0.04–0.62, *p* = 0.0329). Nausea was the most frequent adverse event in both groups (17.5% and 7.5%, respectively), as is shown in Table 4. According to the degree of severity, most of the adverse events were mild in both groups (10/40 in the concomitant group and 2/40 in the tailored group). As well, three patients in the concomitant and one in the tailored therapy group experienced moderate adverse events but without need for special intervention or hospitalization (Table 5).

## 4. Discussion

This is the first randomized clinical trial comparing tailored and concomitant therapy in Croatia. Our study showed that a personalized approach to *H. pylori* treatment offers better results compared to concomitant therapy. Additionally, our clinical trial detected high primary resistance to clarithromycin and metronidazole and an increasing dual resistance rate. The main aim of this study was to elaborate on the optimal therapeutic approach in the treatment of *H. pylori* infection in the Split-Dalmatia region, Croatia, as it is determined that clarithromycin resistance in the Split-Dalmatia County is above 20%, with a relatively low metronidazole resistance rate of 10.2% [22]. Therefore, standard triple therapy is not recommended as a first-line treatment [10]. As stated in Maastricht V guidelines, in areas with high (>15%) clarithromycin resistance, bismuth quadruple or non-bismuth quadruple therapies, primarily concomitant, are recommended [23,24]. Concomitant therapy is now often regarded as the first-line eradication treatment due to its high eradication rate, exceeding 90% in some areas [25]. The standard duration of concomitant therapy is from 10 to 14 days, which includes PPI and three antibiotics: amoxicillin, metronidazole, clarithromycin, which are used for the total period of treatment. This can lead to an increase in antibiotic resistance and abuse of antibiotic use [6]. This can explain the increase in strain resistance that we noted in our trial compared to previous data regarding strain susceptibility tests in our region. Furthermore, as suggested by Maastricht and Toronto guidelines, concomitant therapy is duration-dependent, with a preferable 14-day duration in the first attempt, especially in areas with high clarithromycin resistance [23,26]. Meanwhile, sequential therapy, first introduced as an alternative to triple therapy, was a common first-line treatment in Croatia [18,22]. However, the usage of sequential therapy showed limitations. In areas with high clarithromycin resistance, sequential therapy can be less effective than concomitant therapy [27]. Efficacy of sequential therapy drops down significantly when *H. pylori* strains were clarithromycin-resistant, even down to 70%, as presented by Liou et al. [28]. There is also evidence that sequential therapy is affected by metronidazole resistance [28].

However, as our study has shown, the tailored approach has better efficiency and lower adverse rate incidence compared to the standard empirical approach. This finding has increasing importance in the setting of higher levels of antimicrobial resistance. Recently, eradication regimes are diminishing in their efficacy, and approximately 30–40% of patients require second-line therapy [19,29]. As well, there is a significant problem of second-line and third-line eradication therapy due to, as well, increase in antibiotic resistance. Currently, the Maastricht Consensus Conferences recommends the use of *H. pylori* strain antimicrobial susceptibility testing only after failure of second-line treatment [3,30]. There are, however, findings from a meta-analysis by Chen et al. showing better eradication regimes in most first-line tailored regimes than in the empiric groups, thereby strongly suggesting a tailored approach as an alternative first-line eradication choice [31]. That result clearly incorporates into a wider context of a personalized, individual approach to patient treatment. It underlines the current deficit in the treatment of *H. pylori* as it is the only infectious disease that is treated empirically rather than according to individual strain antimicrobial susceptibility [32,33,34].

As mentioned, our study showed high and increasing resistance to both clarithromycin and metronidazole (37.5% and 17.5%, respectively). Dual resistance to both of these antibiotics is also increasing (10%). These results are highly significant regarding the fact that concomitant therapy can have lower efficacy in areas with high dual resistance or high metronidazole resistance [20,35]. Results of one meta-analysis demonstrated that the eradication rate of concomitant therapy was only 33.3–66.7% for strains with dual clarithromycin-metronidazole resistance [25]. To overcome these problems, other quadruple therapies, such as sequential and hybrid, were proposed. However, there are reports showing an increased prevalence of resistance to quinolone in the last decade, and that problem is actuated by the fact that there is no dose-dependent effect of overcoming quinolone resistance as opposed to metronidazole [36]. In that setting, eradication rates that we achieved in our study both in the intention-to-treat and per-protocol group (92.5% and 100%, respectively) clearly indicate that tailored approach is the only kind of treatment that achieves a high eradication rate whilst at the same time reducing the problem of potential first-line or second-line empirical treatment failure which generates higher future rates of antimicrobial resistance.

The secondary objectives of the study were to determine the tolerability of these therapeutic protocols based on compliance and adverse events occurrence. In all therapeutic regimes, compliance rate could be another potential factor for the eventual failure of eradication treatment. In our study, in both groups compliance rate was more than satisfactory, with no significant difference, although we expected better compliance in the tailored group regarding a smaller number of antibiotics. As we expected, less antibiotic usage resulted in less adverse events. We demonstrated a significantly higher adverse events rate in concomitant than in the tailored group, with nausea being the most common adverse event in both groups. Our results clearly incorporate into a wider group of results that show the beneficial effect of reducing antibiotic intake [12]. There were no differences in specific adverse events among groups. Adverse events were mild according to the degree of severity, and three patients who had moderate events were in the concomitant group, and one was in the tailored group. Furthermore, the tailored group had significantly lower adjusted odds of adverse events.

Although this is the first randomized clinical trial comparing tailored and concomitant therapy in Croatia, our study has few limitations. It is a well-established fact that in vitro susceptibility testing does not always correspond to in vivo eradication, thereby limiting the potential success of eradication in all settings [33]. There is, as well, an increasing problem of healthcare costs whereby the additional cost of sampling and microbiology testing could pose a problem from a healthcare economics standpoint [8,37,38]. Infection with multiple strains of *H. pylori* could influence the success of eradication rates, thus limiting the value of a tailored approach [34]. Our study has some methodological drawbacks as well. It is limited by the small sample size and therefore has a limited statistical significance. It clearly does not represent a multicenter study. We should, therefore, stress that multicenter randomized controlled studies should be a priority in assessing the full value of the tailored approach. Secondly, this study was designed as an open-label one, which may increase the potential risk of bias. Although the majority of similar *H. pylori* clinical trials are open-label, blind-design studies are necessary for avoiding potential bias [39]. Finally, this study was not designed as a non-inferiority one, which may affect its conclusiveness. Thus, a non-inferiority trial should be conducted for further comparison of these two protocols, with a greater sample size. Still, tailored therapy in the era of personalized medicine should be regarded as a potential future approach in clinical practice. Regarding the fact that our region has a clarithromycin resistance rate above 20%, the results of this study may be applicable to regions with a similar problem.

## 5. Conclusions

In conclusion, tailored therapy based on antibiotic susceptibility testing shows a significantly higher eradication rate than the comparable empirical treatment. Therefore, this therapy could help achieve better eradication results and promote a personalized medicine approach to future patients in our region. Regarding the lesser number of antibiotics, fewer adverse events and higher eradication rate, we suggest that tailored therapy could be the first-line treatment option in areas with high clarithromycin resistance. Therefore, we encourage further bigger multi-center studies to investigate the potential of this novel approach.

## Figures and Tables

**Table 1:** The baseline characteristics of the study population.

Parameter	Concomitant Therapy (N = 40)	Tailored Therapy (N = 40)	*p* *
Male gender (N, %)	21 (52.5)	17 (42.5)	0.373 *
Age (years)	61.9 ± 12.7	60.4 ± 13.4	0.628 ^†^
Smoking (N, %)	7 (17.5)	11 (27.5)	0.287 *
Alcohol consumption (N, %)	8 (20.0)	5 (12.5)	0.546 ^‡^
Endoscopic findings			
Gastritis	32 (80.0)	33 (82.5)	0.503 ^‡^
Gastric ulcer	5 (12.5)	5 (12.5)	
Duodenal ulcer	2 (5.0)	0 (0.0)	
Duodenitis	1 (2.5)	2 (5.0)	

* chi-square test. † *t*-test for independent samples. ‡ Fisher’s exact test.

**Table 2:** The clinical outcomes of the study population.

Parameter	Concomitant Therapy (N = 40, %)	Tailored Therapy (N = 40, %)	*p* *
Eradication rate:			
Intention-to-treat (%; 95% CI)	28/40 (70.0; 55.8–84.2)	37/40 (92.5; 85.0–100.0)	0.010
Per-protocol (%; 95% CI)	28/32 (87.5; 76.1–98.9)	36/36 (100.0; /)	0.030
Compliance >80%	34/40 (85.0)	36/40 (90.0)	0.502
Adverse effects	13/40 (32.5)	3/40 (7.5)	0.006 ^†^

* chi-square test. † Fisher’s exact test.

**Table 3:** Antimicrobial resistance in the study population.

Antimicrobial Agent	Strains Tested (N)	Primary Antibiotic Resistance n (%)
Clarithromycin	40	15 (37.5)
Metronidazole	40	7 (17.5)
Levofloxacin	40	2 (5)
Amoxicillin	40	0 (0)
Dual resistance		
Clarithromycin, Metronidazole	40	4 (10)
Clarithromycin, Levofloxacin	40	2 (5)

**Table 4:** The adverse effects of the study population †.

Parameter	Concomitant Therapy ^1^ (N = 40)	Tailored Therapy ^1^ (N = 40)	*p* *
Nausea	7 (17.5)	3 (7.5)	0.311
Stomach pain	1 (2.5)	0 (0.0)	0.998
Skin rash	2 (5.0)	0 (0.0)	0.493
Metallic taste	2 (5.0)	0 (0.0)	0.493
Headache	1 (2.5)	0 (0.0)	0.998
Diarrhea	1 (2.5)	0 (0.0)	0.998
Tongue deposits	1 (2.5)	0 (0.0)	0.998

^1^ Data are presented as a whole number and percentages. * Fisher’s exact test. † Some patients had more than one adverse effect.

**Table 5:** Adverse effects severity analysis.

Adverse effect †	Concomitant Therapy ^1^ (N = 40)	Tailored Therapy ^1^ (N = 40)	*p* *
None	27 (67.5)	37 (92.5)	0.019
Mild	10 (25.0)	2 (5.0)	
Moderate	3 (7.5)	1 (2.5)	

^1^ Data are presented as a whole number and percentages. * Fisher’s exact test. † Severe adverse effects have not been reported.

## Data Availability

The data presented in this study are available on request from the corresponding author. The data are not publicly available because some of the data sets will be used for further research.
